# Imbalance of Chemokines and Cytokines in the Bone Marrow Microenvironment of Children with B-Cell Acute Lymphoblastic Leukemia

**DOI:** 10.1155/2021/5530650

**Published:** 2021-07-22

**Authors:** Fábio Magalhães-Gama, Marlon Wendell Athaydes Kerr, Nilberto Dias de Araújo, Hiochelson Najibe Santos Ibiapina, Juliana Costa Ferreira Neves, Fabíola Silva Alves Hanna, Lilyane de Amorim Xabregas, Maria Perpétuo Socorro Sampaio Carvalho, Eliana Brasil Alves, Andréa Monteiro Tarragô, Olindo Assis Martins-Filho, Andréa Teixeira-Carvalho, Adriana Malheiro, Allyson Guimarães da Costa

**Affiliations:** ^1^Programa de Pós-Graduação em Imunologia Básica e Aplicada, Universidade Federal do Amazonas (UFAM), Manaus, Brazil; ^2^Diretoria de Ensino e Pesquisa, Fundação Hospitalar de Hematologia e Hemoterapia do Amazonas (HEMOAM), Manaus, Brazil; ^3^Programa de Pós-Graduação em Ciências da Saúde, Instituto René Rachou-Fundação Oswaldo Cruz (FIOCRUZ) Minas, Belo Horizonte, Brazil; ^4^Grupo Integrado de Pesquisas em Biomarcadores de Diagnóstico e Monitoração, Instituto René Rachou-FIOCRUZ Minas, Belo Horizonte, Brazil; ^5^Programa de Pós-Graduação em Ciências Aplicadas à Hematologia, Universidade do Estado do Amazonas (UEA), Manaus, Brazil; ^6^Programa de Pós-Graduação em Medicina Tropical, UEA, Manaus, Brazil; ^7^Instituto de Pesquisa Clínica Carlos Borborema, Fundação de Medicina Tropical Dr. Heitor Vieira Dourado (FMT-HVD), Manaus, Brazil; ^8^Escola de Enfermagem de Manaus, UFAM, Manaus, Brazil

## Abstract

In the hematopoietic microenvironment, leukemic cells secrete factors that imbalanced chemokine and cytokine production. However, the network of soluble immunological molecules in the bone marrow microenvironment of acute lymphoblastic leukemia (ALL) remains underexplored. Herein, we evaluated the levels of the immunological molecules (CXCL8, CCL2, CXCL9, CCL5, CXCL10, IL-6, TNF, IFN-*γ*, IL-17A, IL-4, IL-10, and IL-2) in the bone marrow plasma of 47 recently diagnosed B-cell acute lymphoblastic leukemia (B-ALL) patients during induction therapy using cytometric beads arrays. The results demonstrated that B-ALL patients showed high levels of CXCL9, CXCL10, IL-6, and IL-10 at the time of diagnosis, while at the end of induction therapy, a decrease in the levels of these immunological molecules and an increase in CCL5, IFN-*γ*, and IL-17A levels were observed. These findings indicate that B-ALL patients have an imbalance in chemokines and cytokines in the bone marrow microenvironment that contributes to suppressing the immune response. This immune imbalance may be associated with the presence of leukemic cells since, at the end of the induction therapy, with the elimination and reduction to residual cells, the proinflammatory profile is reestablished, characterized by an increase in the cytokines of the Th1 and Th17 profiles.

## 1. Introduction

B-cell acute lymphoblastic leukemia (B-ALL) is characterized by an abnormal proliferation of lymphoblasts in the bone marrow and represents the most common childhood cancer in the world [1, 2]. Immune system imbalance has been demonstrated to be involved in the pathogenesis of ALL [3]. Studies have reported an imbalance in the concentrations of soluble immunological molecules, including chemokines and cytokines, which can contribute to a state of cellular immunosuppression and promotes the survival and proliferation of leukemic cells [4–7].

This imbalance has also been observed in acute myeloblastic leukemia (AML) and represents a potential mechanism for evading immune surveillance [8–11]. The interaction of the chemokines and cytokines produced in the tumor microenvironment plays an important role during the neoplastic process [12]. Cancer cells have been shown to “hijack” the immunological molecule networks to support disease progression [13, 14]. In the context of ALL, the imbalance in the chemokine and cytokine network has been reported mainly in peripheral blood, while in the bone marrow microenvironment, it remains underexplored [5, 6, 15].

Bone marrow is the main site of control of the proliferation and dissemination of leukemic cells. Therefore, the levels of soluble immunological molecules in peripheral blood do not precisely reflect the immune status of the disease, and this indicates the need for studies on the complex immune network in the leukemic microenvironment. Herein, we evaluate the chemokine and cytokine levels in bone marrow aspirates of children that had been recently diagnosed with B-ALL during induction therapy. Our aim was to characterize the profile of these molecules in the leukemic microenvironment. The data presented in this study demonstrate that B-ALL patients have an imbalance in chemokine and cytokine levels that contributes to an immunoregulatory profile that is favorable to disease progression.

## 2. Materials and Methods

### 2.1. Ethics Statement

This study was submitted to and approved by the Ethics Committee at Fundação Hospitalar de Hematologia e Hemoterapia do Amazonas (HEMOAM), under protocol registration number #739.563/2014. Before the inclusion of all patients in the study, all the respective parents or legal guardians read and signed the informed consent form. The study was carried out in accordance with the principles of the Declaration of Helsinki and Resolution 466/2012 of the Brazilian National Health Council, which relates to research involving human participants.

### 2.2. Study Population

Between August 2014 and March 2018, a total of 47 pediatric patients were included in this study carried out at the HEMOAM Foundation, which is the reference center for diagnosis and treatment of hematological diseases in Manaus, Amazonas state, Brazil. All 47 patients in the study were recently diagnosed with B-ALL, were of either sex (34 males and 13 females), and had a median age of 5 years; interquartile range (IQR) = 3–10. The diagnosis was performed according to the classification criteria and guidelines of the World Health Organization [16]. The clinical and hematological characteristics of B-ALL patients include age, sex, total leukocytes, the count of blast cells in bone marrow and peripheral blood, neutrophil count, lymphocyte count, monocyte count, hemoglobin level, and platelet count. All the clinical and hematological characteristics are summarized in Table 1.

### 2.3. Treatment Regimen

All B-ALL patients underwent induction therapy (according to the protocol and guidelines found in the Brazilian Group for Treatment of Childhood Leukemia, version 2009), which is an intensive chemotherapy stage of fundamental importance for the prognosis of patients, and whose objective is to achieve complete clinical remission in 4 weeks. The treatment regimen includes the drugs prednisone, dexamethasone, vincristine, daunorubicin, L-asparaginase, and MADIT (intrathecal methotrexate, cytarabine, and dexamethasone) [17]. At the end of the induction therapy (D35), 14 patients achieved remission (reference value: <0.001% blasts, flow cytometry).

### 2.4. Biological Sample Collection

The bone marrow samples of B-ALL patients were obtained by iliac crest aspiration at two consecutive time points during the induction therapy as follows: D0 (Day 0, day of diagnosis) and D35 (Day 35, the end of the induction therapy). After collection, the samples were transferred to EDTA vacuum tubes (BD Vacutainer® EDTA K2) and submitted to centrifugation at 900x g for 15 minutes. Subsequently, the supernatants were collected and immediately stored at −80°C until processing for analysis of the soluble immunological molecules.

### 2.5. Quantification of Chemokines and Cytokines

The concentration of soluble immunological molecules, including chemokines and cytokines (CXCL8, CCL2, CXCL9, CCL5, CXCL10, IL-6, TNF, IFN-*γ*, IL-17A, IL-4, IL-10, and IL-2), was quantified using cytometric bead arrays (BD™ Human Chemokine and BD™ Human Cytokine Th1/Th2/Th17 kits, BD Biosciences, San Diego, CA, USA), according to the manufacturer's instructions. Samples were acquired in a FACSCanto II (BD Biosciences, San Jose, CA, USA), and the FCAP-Array software v3 (BD Biosciences, San Jose, CA, USA) was used for data analysis. Data were reported in picograms per milliliter (pg/mL) concentrations, according to the standard curves provided in the kits.

### 2.6. Conventional Statistical Analysis

Comparative analyses between the time points of induction therapy (D0 and D35) were performed using a Wilcoxon matched-pairs signed ranks test. In all cases, significance was considered at *p* < 0.05. GraphPad Prism, version 8.0.1 (GraphPad Software, San Diego, CA, USA), was used for the statistical analyses.

### 2.7. Correlation Network Analysis

Correlation networks were assembled to evaluate the multiple associations among the soluble immunological molecules in the B-ALL patients. The association between the quantitative levels of chemokines and cytokines was determined using the Spearman correlation coefficient in GraphPad Prism, version 8.0.1 (GraphPad Software, San Diego, CA, USA), and statistical significance was considered only if *p* < 0.05. After performing the correlation analysis between immunological molecules, a database was created using the Microsoft Excel program. Then, the significant correlations were compiled using the open-source Cytoscape software, version 3.0.3 (National Institute of General Medical Sciences, Bethesda, MD, USA). Chemokine and cytokine networks were constructed using circular layouts in which each molecule is represented by a globular node. The correlation indices (*r*) were used to categorize the correlation strength as negative (*r* < 0), moderate (0.36 ≥ *r* ≤ 0.68), and strong (*r* > 0.68), which were represented by connecting edges, as proposed by Taylor (1990) [18]. Correlation matrices were also developed using the Microsoft Excel program. Correlation matrices display significant association (*p* < 0.05) between immunological molecules pairs based on the rank indices, which are tagged by color keys, ranging from −1.0 to 1.0 to underscore the correlation strength, according to the color key provided. Cytoscape, Microsoft Excel, and PowerPoint Software were used for the graphics.

## 3. Results

### 3.1. Follow-Up of the Chemokine and Cytokine Levels in the Bone Marrow Microenvironment of B-ALL Patients during Induction Therapy

**Figure 1:**
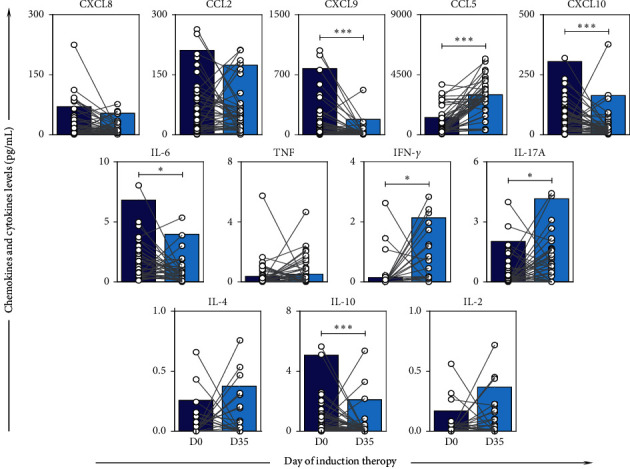
Follow-up of the chemokine and cytokine levels in the bone marrow microenvironment of B-ALL patients on D0 and D35 of induction therapy. The immunological molecules (CXCL8, CCL2, CXCL9, CCL5, CXCL10, IL-6, TNF, IFN-*γ*, IL-17A, IL-4, IL-10, and IL-2) levels were measured in bone marrow plasma from B-ALL patients on D0 (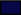) and D35 (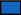) using cytometric bead arrays as described in the Methods section. Data are expressed as mean with SEM in picograms per milliliter (pg/mL) concentration. Statistical analyses were performed using the Wilcoxon matched-pairs signed ranks test. The results are presented using bar plots, in linear scale. Significant differences (*p* < 0.05) are highlighted by connecting lines and asterisks (^*∗*^).

The analysis of the production dynamics of chemokines and cytokines in the bone marrow microenvironment was performed on D0 and D35 in order to evaluate the behavior of these immunological molecules at the beginning and the end of induction therapy. The results demonstrated high levels of the chemokines CXCL9, CXCL10, and the cytokines IL-6 and IL-10 at the time of diagnosis (D0) and a significant decrease at the end of the induction therapy (D35), while the molecules CCL5, IFN-*γ*, and IL-17A displayed a significant increase (Figure 1).

### 3.2. Networks of Chemokines and Cytokines in the Bone Marrow Microenvironment of B-ALL Patients during Induction Therapy

**Figure 2:**
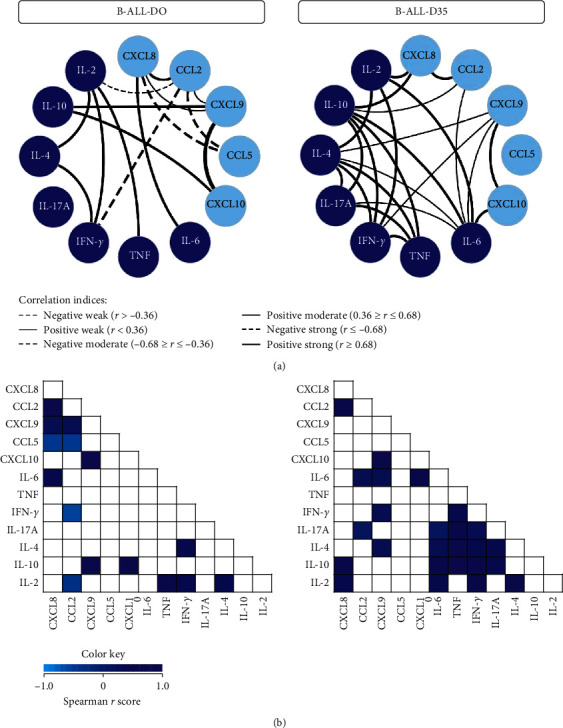
Networks of chemokines and cytokines in the bone marrow microenvironment of B-ALL patients on D0 and D35 of induction therapy. Customized chemokine and cytokine network layouts were assembled to identify the relevant association among immunological molecules in the bone marrow microenvironment of B-ALL patients on D0 and D35. Colored nodes are used to identify the chemokines (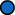) and cytokines (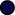) (a). Significant Spearman's correlations at *p* < 0.05 were represented by connecting edges to highlight positive (strong (*r* ≥ 0.68 = thick continuous line), moderate (0.36 ≥ *r* ≤ 0.68 = thinner continuous line), or weak (*r* < 0.36 = thin continuous line)) and negative (strong (*r* ≤ −0.68 = thick dashed line), moderate (−0.68 ≥ *r* ≤ −0.36 = thinner dashed line), or weak (*r* > −0:36 = thin dashed line)) as proposed by Taylor (18). Correlation matrices were also developed. Correlation matrices display significant association (*p* < 0.05) between immunological molecule pairs based on the rank indices, which are tagged by color keys (different shades of blue), ranging from −1.0 to 1.0 to underscore the correlation strength (b). (a) Networks of chemokines and cytokines. (b) Correlation matrices.

The construction of integrative networks for the samples of B-ALL patients was performed to demonstrate the interaction of chemokines and cytokines in bone marrow on D0 and D35 (Figure 2(a)). The results showed that the B-ALL patients on D0 had a restricted network of chemokines/cytokines, with a small number of neighborhood connections, including characteristic negative correlations among the immunological molecules CXCL8/CCL2 and CCL5/IFN-*γ*/IL-2. While on D35, it is possible to observe that the B-ALL patients lost the negative correlations shown on D0 and presented a distinct change to a proinflammatory profile, with an increase in the interactions between soluble immunological molecules, including T helper-associated cytokines. Finally, correlation matrices were developed in order to illustrate the strength of correlation between immunological molecules and provide a more simplified view of the networks (Figure 2(b)).

### 3.3. Networks of Blasts and Chemokines/Cytokines in the Bone Marrow Microenvironment of B-ALL Patients at the Time of Diagnosis

**Figure 3:**
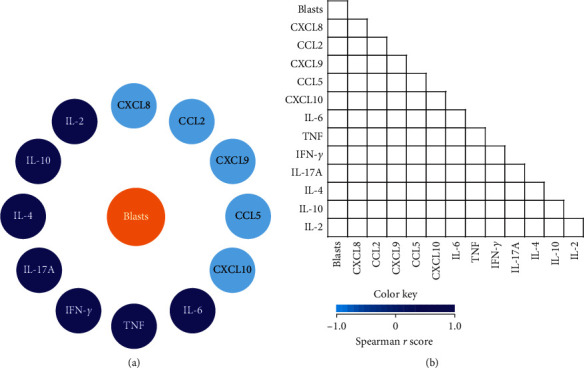
Networks of blasts and chemokines/cytokines in the bone marrow microenvironment of B-ALL patients on D0 of induction therapy. Customized blast network layouts were assembled to investigate their association with immune molecules in the bone marrow microenvironment of B-ALL patients on D0. Colored nodes are used to identify the blasts (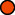), chemokines (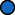), and cytokines (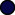) (a). Correlation matrices display significant associations (*p* < 0.05) between blasts and immunological molecules based on the rank indices, which are tagged by color keys (different shades of blue), ranging from −1.0 to 1.0 to underscore the correlation strength (b). (a) Network of blasts and chemokines/cytokines. (b) Correlation matrices.

In order to investigate the correlation between the levels of soluble immunological molecules with the number of blasts at the time of diagnosis, an integrative network was constructed (Figure 3(a)) together with the correlation matrices (Figure 3(b)). However, our data did not show correlations between the levels of chemokines/cytokines and blast count on D0.

## 4. Discussion

The signaling of soluble immunological molecules is essential for the homeostasis of the hematopoietic microenvironment and promotes complex interactions between hematopoietic cells and the surrounding stromal cells [19, 20]. However, leukemic cells secrete factors that disrupt healthy bone marrow niches and reprogramme and transform them into “leukemic niches” [21]. This event results in a disruption of the balanced production of chemokines and cytokines and provides an immunosuppressive and permissive microenvironment for leukemic progression [22–25].

The immunosuppression process is evidenced by the presence of the cytokine IL-10, which plays an important role in the tumor microenvironment by decreasing the cytotoxic immune response [26, 27]. In addition, high levels of IL-10 in serum and the tumor microenvironment have often been correlated with a worse prognosis in solid neoplasms and hematological malignancies, including ALL [7, 15, 28–32]. In our data, it was observed that the B-ALL patients had high levels of IL-10 at the time of diagnosis (D0) (Figure 1).

Likewise, an increase in CXCL9 and CXCL10 levels was observed on D0. The chemokines CXCL9 and CXCL10, together with CXCL11 correspond to selective ligands for CXCR3. Usually, the CXCL9-10-11/CXCR3 axis regulates the migration and activation of immune cells and exhibits an antitumor effect [33]. However, investigations have shown that the CXCL9-10-11/CXCR3 axis is able to contribute to the proliferation and metastasis of cancer cells [34–36]. In leukemia, this was observed in a study involving pediatric patients with ALL, with CXCL10 inducing chemotaxis of leukemic cells that expressed CXCR3, and also decreased chemotherapy-induced apoptosis in the leukemic cells CXCR3^+^ [36]. It is important to highlight that, in the tumor microenvironment, CXCL9-10-11 chemokines are secreted mainly by endothelial cells, fibroblasts, and cancer cells in response to IFN-*γ*, which are synergistically increased by TNF [37, 38]. Interestingly, our data did not show high levels of IFN-*γ* and TNF; on the contrary, low levels of both cytokines were observed on D0. This supports a potential negative effect of CXCL9 and CXCL10 on ALL.

Another chemokine that presented a potential reversal of the microenvironment mechanism in favor of leukemic cells is IL-6. It has been reported that IL-6 acts as an essential agent, exerting different protumorigenic activities that intrinsically act on cancer cells through numerous mediators that support the proliferation, survival, and dissemination of these cells and that extrinsically act on cells that make up the tumor microenvironment, thus supporting angiogenesis and evasion of immune surveillance [39–42]. Furthermore, studies have shown that a concentration of high IL-6 may be indicative of a poor prognosis in patients with solid or hematologic malignancies [43, 44]. In our study, higher concentrations of IL-6 were also observed on D0.

Following these findings, through the monitoring of patients, a decrease in the levels of CXCL9, CXCL10, IL-6, and IL-10 was observed at the end of the induction therapy (D35). This demonstrates that chemotherapy treatment can correct the cellular immunosuppression presented by these patients on D0 since it is responsible for the elimination of leukemic cells. In addition, these results indicate that these molecules may be involved in the pathogenesis of ALL.

Induction therapy also corrected levels of IFN-*γ*, which is a key cytokine of the Th1 profile. This is in accordance with previous studies that reported an increase in the percentage of IFN-*γ* producing helper T cells at the end of induction therapy [5]. In addition, it has been reported that leukemic cells of acute myeloblastic leukemia (AML) secrete soluble factors that prevent T cells from secreting cytokines related to the Th1 profile [45]. This finding supports our results since it is expected that on D35, there will be no more leukemic cells in the medullary compartment.

IL-17A, the key cytokine of the Th17 profile, also showed a significant increase in D35. This demonstrates that, as observed with IFN-*γ*, which showed an increase at the end of induction therapy, higher levels of IL-17A may indicate the recovery of the inflammatory response with the elimination of leukemic cells. These findings are reinforced by the increased levels of CCL5, a chemokine that under normal conditions plays an important role in recruiting a variety of leukocytes, including macrophages, natural killer cells, and helper T cells [46, 47].

The analysis of the integrative network of chemokines and cytokines also demonstrated important alterations during induction therapy. On D0, B-ALL patients exhibited a network of immunological molecules that were characterized by a limited number of interactions, with negative correlations between CXCL8 and CCL5, and among CCL2 and CCL5 and IFN-*γ* and IL-2 (Figure 2). Studies have demonstrated that the positive regulation of CXCL8, together with CCL2, may contribute to the establishment of a malignant microenvironment that is favorable to leukemic cells in B-ALL [48]. Moreover, the bone marrow plasma levels of CXCL8 in B-ALL patients on diagnosis were significantly higher than those in the healthy controls [48]. Thus, the negative correlations of CXCL8 and CCL2 with CCL5, IFN-*γ*, and IL-2 may be indicative of an immune balance, with the CXCL8/CCL2 axis overlapping and reducing the interactions between immunological molecules related to the establishment of a cytotoxic immune response. This finding is reinforced by the moderate and strong correlations established among CXCL9, CXCL10, and IL-10, which are molecules that have a negative effect on ALL.

**Figure 4:**
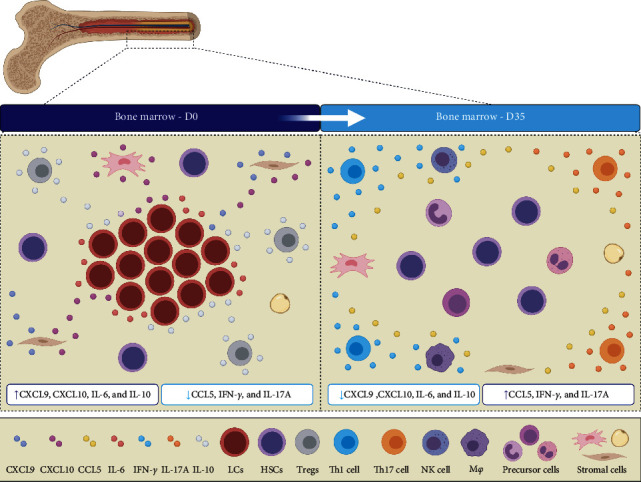
Bone marrow microenvironment of B-ALL patients on D0 and D35 of induction therapy. Schematic presentation of the possible bone marrow microenvironment during induction therapy based on the main findings of the study. It is noteworthy that the cell repertoires represented in the figure were not evaluated in the present study. LCs: leukemic cells; HSCs: hematopoietic stem cells; Treg: regulatory T cells; Th1: T helper 1 cells; Th17: T helper 17 cells; NK: natural killer cells; M*φ*: macrophage.

On D35, the B-ALL patients exhibited a network that was characterized by the recovery of the proinflammatory response. This is composed of greater interactions among the immunological molecules of the different response profiles (Th1, Th2, and Th17 or a mixed pattern). Furthermore, it is important to highlight the absence of negative correlations among CXCL8/CCL2 and CCL5/IFN-*γ*/IL-2 and moderate and strong correlations among CXCL9, CXCL10, and IL-10. This immune imbalance may be associated with the presence of leukemic cells in the bone marrow compartment, since on D35, with the elimination or expressive reduction of the number of blasts observed on D0, immune restoration is observed, and the imbalance of the chemokines and cytokines network is corrected (Figure 4).

Notwithstanding, this study presented some limitations. Since bone marrow aspiration is a quite invasive procedure, the Ethics Committee at the HEMOAM Foundation did not allow this procedure to be performed in healthy children, which ended up making it impossible to use a control group for comparative purposes in the analysis. In addition, cellular repertoires could not be evaluated due to the scarce amount of bone marrow material obtained.

## 5. Conclusions

In summary, our data demonstrated that the elevated levels of CXCL9, CXCL10, IL-6, and IL-10 and the moderate and strong correlations displayed by CXCL9, CXCL10, and IL-10, together with the negative correlations of CXCL8 and CCL2, may be related to the suppression of the Th1 profile on D0, which contributes synergistically to an immune system imbalance in B-ALL. Furthermore, induction therapy has been shown to correct abnormal levels of chemokines and cytokines on D35, thus decreasing levels/correlations of CXCL9, CXCL10, IL-6, and IL-10 and increasing concentrations of CCL5, IFN-*γ*, and IL-17A, and indicates the recovery in levels and interactions of the Th1, Th2, and Th17 profiles. However, it is important that additional studies should be carried out regarding the mechanisms involved in cellular immunosuppression and the sequestration of the chemokine and cytokine network in the leukemic microenvironment.

## Figures and Tables

**Table 1:** Characteristics of B-ALL patients at D0.

Characteristics	B-ALL (*n* = 47)
Age (years), median (IQR)	5 (3–10)
Sex, (male/female)	34M/13F
Total leukocytes (×10^3^/*μ*L), median (IQR)	56.735 (41.865–76.825)
Marrow blast (ABS), median (IQR)	47.964 (28.483–64.920)
Blood blast (ABS), median (IQR)	7.239 (995–8.278)
Neutrophils (×10^3^/*μ*L), median (IQR)	0.43 (0.18–0.98)
Lymphocytes (×10^3^/*μ*L), median (IQR)	3.10 (2.25–4.29)
Monocytes (×10^3^/*μ*L), median (IQR)	0.11 (0.00–0.21)
Hemoglobin (g/dL), median (IQR)	8.3 (3.7–9.7)
Platelets (×10^3^/*μ*L), median (IQR)	54 (28–101)

B-cell acute lymphoblastic leukemia; ABS, absolute; IQR, interquartile range. Reference values: leukocytes: 5.2–12.4 × ×10^3^/*μ*L; neutrophils: 1.9–8 × ×10^3^/*μ*L; lymphocytes: 0.9–5.2 × ×10^3^/*μ*L; monocytes: 0.16–1 × ×10^3^/*μ*L; hemoglobin: 12–18 g/dL; platelets: 130–140 × ×10^3^/*μ*L.

## Data Availability

The data used to support the findings of this study are included within the article.
